# The gift of freedom? Exploring the realities of rescue from child trafficking in Ghana

**DOI:** 10.1080/03906701.2024.2431909

**Published:** 2024-12-01

**Authors:** Bernard Koomson

**Affiliations:** School of Sociology, Politics and International Studies, University of Bristol, Bristol, UK

**Keywords:** Child trafficking, freedom, NGOs, rescue, young adults

## Abstract

This paper is a critical reflection on the rescue and reintegration regime associated with the child trafficking intervention in Ghana. The paper acknowledges efforts by anti-trafficking NGOs (Non-Governmental Organisations) to address conditions of poverty, child/youth economic exploitation, and human rights abuses. However, it also observes that these interventions have had very little impact for some of the beneficiaries. Drawing on ethnographic research with young adults considered as trafficked victims by a child rights NGO in Ghana, the paper demonstrates how reintegrated young adults are still confronted with the socio-structural factors (residential instability, economic exploitation, physical abuse, and attendance difficulties at school) that preceded their transportation to riverine communities for work in fishing. The paper attributes these post-rescue challenges to the inability of the existing remedial regime to address the structural problems that underline children and young adults’ economic mobility. The paper argues for a more nuanced approach to child trafficking intervention in Ghana.

## Introduction

Within child trafficking intervention, we work in communities along the Volta Lake which is the largest man-made lake in the world. The fishers look for cheap labour because they know children living within coastal communities mostly support their parents, know how to swim, mend nets, and engage in other fishing activities. So, they come and recruit them, send them to communities along the Volta Lake where they work for years, unpaid and are not properly catered for in terms of health and their wellbeing. This is the kind of slavery we fight by rescuing, rehabilitating, and providing medium term care for the victims. (NGO Officer, Winneba)

Economic mobility among Ghana’s under-18 population has been a constant feature of the country’s migration trends (Koomson et al., 2021; Kwankye et al., 2009; Okyere, 2017; Whitehead et al., 2007). As a bulwark against economic hardships and contribution to household income, teenage labour has remained vital to the homes and lives of lower middle income since the colonial era (Boakye-Boaten, 2010; Hashim & Thorsen, 2011). In its shape and form, it entails manual and artisanal labour within the indigenous fishing sector, mining, and farming (Agbenya, 2009; Okyere, 2017). While activities within these sectors had hitherto raised few eyebrows, recent developments within Ghana’s child protection space have led to the problematisation (within policy) of children’s mobility for work, especially in fishing and mining. Antitrafficking and child rights NGOs (Non-Governmental Organisations) in Ghana, working in partnership with state agencies, have drawn attention to the hazardous dimensions of fishing for children by rescuing children (mostly boys) brought to riverine communities from coastal communities. As evident in the opening quotation, NGO officials employ the language of human trafficking and slavery to articulate concerns that characterise under-18s’ work in fishing, creating an urgent need for rescue or child saving. This model of intervention, which draws inspiration from the Palermo Protocol and its contextualised legal framework (in Ghana’s case the Human trafficking Act, 2005), as well as the United States State Department efforts against ‘slavery’ in the Global South (Okyere, 2022; Ramachandran, 2019), is confronted by the socioeconomic inequities and cultural value systems that have long preserved traditions of under-18s’ economic migration to fishing communities.

This article will demonstrate how the rescue and reintegration of children considered trafficked may not necessarily eradicate concerns of exploitation and abuse that characterise the hazardous framing of children’s mobility for work in fishing. Through narrative interviews and ethnographic observations, the paper illuminates the multiplicity of factors (socio-economic, cultural, and legal) that combine to impact the lives of young adults after reintegration . This paper is by no means an attempt to dismiss the efforts of child rights NGOs working within human trafficking intervention in Ghana; rather it draws attention to the various contradictions and illusions of the rescue logic (freedom) prosecuted by NGOs, while providing alternative policy and intervention options. It tracks the daily lives of young adults who have been rescued and reintegrated by NGOs to examine assumptions that surround rescue operations and their related consequences. Before outlining this paper’s main objectives and arguments, I will briefly clarify two intersecting concepts (child/children and young adults) that are mainly employed in the paper. By ‘children’, I mean persons below the age of 18 years as clearly indicated in Ghana’s Children Act, 1998 (Act 560), while ‘young adults’ refers to persons between the ages of 16 and 22 years as most child rights NGO categorise in their interventions. The paper utilises these two intersecting terms because whereas rescues are conducted for children, post-rescue interventions overlap into the period of young adulthood.

The paper’s main argument is that the sheer removal of children from economic activities considered exploitative and hazardous within specific localities does not necessarily tranquilise and romanticise children’s lived experience within their communities of origin. The first section of the paper sets out the background by providing the raison d’etre of the study. The second part of the paper provides socio-legal basis for rescues in anti-trafficking intervention and is intended to be a review of the complexities of NGO’s antitrafficking/slavery activities. The third section discusses child trafficking and its attendant policies in Ghana while the fourth section links kinship fostering to the discussions on child trafficking policy and intervention. The fifth part of the paper provides an overview of the research context and methods, and this is followed by an exploration of the concept of freedom and the realities of post-rescue life in the sixth section. The seventh part entails a critical synthesis of literature and key findings arising from the ethnographic data presented in this paper. The last section details the conclusion and alternative suggestions to current approaches employed by NGOs.

## Law, NGOs, and rescue: a critical analysis of Ghana’s legal and policy framework on anti-trafficking

While the power to conduct rescues rest within the ambit of law enforcement agencies in Ghanaian law, child rights NGOs and their development partners have led the charge in recent years. This is in part traceable to the global child protection regime which mushroomed in the latter part of the twentieth century. For instance, Ghana’s ratification of the United Nations Convention on the Rights of the Child (UNCRC) in 1990 precipitated the enactment of the Children’s Act, 1998 (Act 560) (Twum-Danso, 2008). The Children’s Act, 1998 (Act 560) subsumed laws relating to children’s welfare and protection against harm/abuse, subsequently serving as the unified reference underlining successive child protection policies. Consequently, for the first time within Ghana’s legal framework, laws on children’s welfare and protection, which were hitherto dispersed throughout several statutes, were brought together into a single codified document that embodied all legal issues relating to children (Twum-Danso, 2008; Woll, 2000). Thus, the Children’s Act, 1998 (Act 560) emerged as the major legal derivative of the UNCRC (United Nations Convention on the Rights of Children), upholding the rights of children against any form of activity and practice considered detrimental to their physical and psychosocial wellbeing, while informing the jurisprudence of subsequent legislations such as Juvenile Justice Act, 2003 (Act 653); the Gender and Children Policy (2002); the Early Childhood Care and Development Policy; and lately the Human Trafficking Act, 2005 (Act 694).

The Children’s Act (1998) follows the UNCRC and its underpinning Western-influenced chronological or calendar age construction of childhood. Section 1 of the Act defines children as persons below 18 years. Furthermore, the minimum age for the engagement of a person into employment is pegged at 15 years, while individuals can only engage in hazardous labour when they attain 18 years of age. Section 91 (1) of the Children’s Act, 1998 (Act 560) indicates that ‘the minimum age for the engagement of a person in hazardous work is 18 years’. Hazardous labour is however described as work that poses a danger to the health, safety, and morals of a person, and is listed in Section 91 (3) of the Children’s Act, 1998 (Act 560) as follows:

(a) going to sea; (b) mining and quarrying; (c) porterage of heavy loads; (d) manufacturing industries where chemicals are produced or used; (e) work in places where machines are used; and (f) work in places such as bars, hotels and places of entertainment where a person may be exposed to immoral behaviour.

Additionally, the Children’s Act, 1998 (Act 560) prohibits the engagement of under-18s in exploitative labour. Section 87 (2) of the Act particularly indicates that ‘labour is exploitative of a child if it deprives the child of its health, education or development’. Implicitly, persons under 18 years (children) are declared unfit for the activities in section 91 (3) of the Children’s Act, 2005 (Act 560), given the effects on children’s health and education, consequently legitimising the grounds for their immediate removal from such economic activities.

Nevertheless, the Act’s (Act 560) definition of who a ‘child’ is, rejects indigenous Ghanaian constructions of childhood. Given that Ghanaian ethnic groups define childhood and adulthood through rites of passage, biological maturity, and other social factors (Koomson & Abdulai, 2021; Twum-Danso, 2016); there is an apparent discord between traditional notions of childhood and the country’s legal provisions of childhood (Laird, 2002). Another area where globally derived standards have received wholesale adoption is the Palermo Protocol (2000) through the enactment of Ghana’s Human Trafficking Act, 2005 (Act 694). Human trafficking is thus defined to reflect the Palermo Protocol:

The recruitment, transportation, transfer, harbouring, trading or receipt of persons for the purpose of exploitation within and across national borders by (a) the use of threats, force or other forms of coercion, abduction, fraud, deception, the abuse of power or exploitation of the vulnerability, or (b) giving or receiving payments and benefits to achieve consent.

By so doing, the definitional problems of the Palermo Protocol are transplanted into the country’s legal framework; instead of an actual definition of exploitation, a list of examples is offered (O’Connell Davidson, 2013) in Section 2 of the Act (Act 694) as follows:

Exploitation shall include, at a minimum, the exploitation of the induced prostitution or other forms of sexual exploitation, forced labour or services, slavery or practices similar to slavery, servitude, or the removal of organs.

Moreover, the Act (Act 694) affirms the definition of a child stipulated within the Children’s Act, 1998 (Act 560) by pegging the threshold for the attainment of adulthood at 18 years in article 3(d). There are attempts by the Human Trafficking Act (Act 694) to become more friendly to indigenous Ghanaian childhood through its provision on the concept of ‘placement’ (placing one’s biological child with others for care). In Section 1(3), the Act states that: ‘Placement for sale, bonded placement, temporary placement, placement as service where exploitation by someone else is the motivating factor shall also constitute trafficking’. Even though Lawmakers and anti-trafficking practitioners argued during the course of the field work that Section 1(3) exist to avert wholesale abuse of the practice of kinship fostering as an important socio-cultural provision for alternative child care (which is in fact endorsed by the Children’s Act, 1998, Act 560), the counterfactual is also the case as Section 1(3) seems to have led to the wholesale delegitimising of the practice of child placement or fostering by anti-trafficking practitioners (Koomson & Abdulai, 2021; Okyere et al., 2021). For instance, the specification of various forms of placement: (i) ‘bonded placement’ for offsetting debts; (ii) ‘temporary placement’ for a limited period of exploitation; and (iv) ‘placement as a service’ for exploitative purposes where economic activities of the child are not remunerated, provides a very ambiguous and low-hanging threshold for the conflation of fostering with trafficking and slavery by abolitionists (particularly child rights NGOs), within Ghana’s anti-trafficking/slavery intervention space (Koomson et al., 2021) who draw additional inspiration from other instruments such as the Victims of Trafficking and Violence Protection Act (VTVPA) of 2000 by the United States. To be specific, it only requires one to prove that children have been moved, placed, and utilised by persons other than their biological parents in any of the exploitative activities listed in Sections 2 and 91(3) of the Human trafficking Act, 2005 (Act 694) and Children’s Act, 1998 (Act 560) respectively. Furthermore, the Act in keeping with the content of the Palermo Protocol in Article 3(C) dismisses a generational norm of children’s willing contribution to their household income, which may sometimes entail their movement to other communities for work. Specifically, the Act stipulates that children’s consent in trafficking cannot be used as an alibi of defence. This is clearly stipulated in Section 1 (4):

Where children are trafficked, consent of the child or his/her parents or guardians cannot be used as a defence in prosecution under this Act, regardless of whether or not there is evidence of abuse of power, fraud or deception on the part of the trafficker or whether the vulnerability of the child was taken advantage of.

The use of the phrase ‘regardless of whether or not there is evidence,’ coupled with the ambiguity around child placement means the Ghanaian law does not require the establishment of the evidence of ‘means’ in respect of under-18s when a suspected case of trafficking is determined. Consequently, under-18s’ voluntary economic migration to riverine communities has been problematised by NGOs and their development partners in numerous studies (ILO/IPEC, 2013; Singleton et al., 2016; WFF 2014) even though children and young adults independently move or migrate to work for several reasons (Koomson et al., 2021)^2014^. In this regard Kassa and Abebe () have argued that mainstream child rights discourses and policies misinterpret informal child fostering and relocation practices as problematic and illegal, leading to interventions that criminalise childhood and family poverty. The ensuing framing of children’s economic mobility, hinges on western-centric neoliberal logic of romantic childhood and fails to acknowledge the diversity of childhoods within specific socio-cultural contexts (Abebe, 2019; Koomson & Abdulai, 2021; Twum-Danso, 2016).

Apart from Ghana’s strict adherence to the UNCRC and Palermo Protocol, there exists a host of instruments of global governance such as the US State Department’s Trafficking in Persons (TIP) Report, which annually assess Ghana’s (and other countries in the Global South) efforts in addressing human trafficking adequately in accordance with standards prescribed by the VTVPA of 2000. The VTVPA summarises a host of concerns under a single title (slavery), including forced labour, child labour, labour trafficking, sex trafficking and involuntary servitude (Merry, 2006). Chuang (2013) has described as ‘exploitation creep’, the way the US-led antitrafficking agenda rhetorically conflates hitherto legally distinct concepts of trafficking, slavery, and forced labour to stimulate public outrage, media coverage, and donor support. Accordingly, the concept of ‘modern-day slavery’ as espoused in the VTVPA and its consequential TIP Reports, has a distinctly American referent (Ramachandran, 2019). As indicated by the secretary of state, Hillary Clinton, the TIP Reports embody the United States’ sustained commitment to fighting traffickers no matter where they may be, because fighting slavery and standing up for human rights is part of the identity of the United States (USDS, 2011). In this regard, the US has been described as the ‘global sheriff’ (Chuang, 2006) that plays an oversight role in keeping states such as Ghana in check with conditionalities of donor funding (Okyere, 2022).

The ensuing legal and policy climate provides fertile grounds for NGOs to thrive, as several of them mushroomed overnight to champion the course of children’s rights and anti-trafficking in Ghana (Lawrance, 2010). The Human Trafficking Act, 2005 (Act 694) valorise collaborations between State agencies and NGOs by stating clearly that cases of suspected trafficking can be reported to NGOs. This prevailing context affects how NGOs, especially those who receive US funding, undertake rescue and other post-rescue interventions. Specifically, these NGOs utilise the term ‘child slaves’ to describe children working in the indigenous fishing industry (Okyere et al., 2021). The description of children as slaves within Ghana’s media space evokes emotive and swift reactions in a way that is almost impossible to attain when the term trafficking is utilised more broadly. Given Ghana’s historical links with the transatlantic slave, it is needless to say that a reframing of the concept of trafficking as a form of slavery would gain public sympathy and justify any form of rescue operation that infringes on the rights of communities along the Lake (Okyere et al., 2021). My fieldwork, discussed in the sixth section of this paper, underscores the disconnect between the intent and promise of NGOs rescue and the post-rescue experiences of young adults.

In this prevailing context, a very low threshold has been set for the identification of child trafficking primarily because policymakers do not accept that a person under the age of 18 is capable of consenting to his or her movement for work and other activities that may be considered exploitative, as Hashim (2006) and Bastia (2005) have both identified. The ILO Convention No. 182, commonly referred to as the Worst Forms of Child Labour Convention, is yet another notable source to which abolitionists have frequently turned in attempting to define child slavery. Article 3 of Convention 182 defines WFCL as:

(a) all forms of slavery or practices similar to slavery, such as the sale and trafficking of children, debt bondage and serfdom and forced or compulsory labour, including forced or compulsory recruitment of children for use in armed conflict; (b) the use, procuring or offering of a child for prostitution, for the production of pornography or for pornographic performances; (c) the use, procuring or offering of a child for illicit activities, in particular for the production and trafficking of drugs as defined in the relevant international treaties; and (d) work which, by its nature or the circumstances in which it is carried out, is likely to harm the health, safety or morals of children. (Article 3, ILO Convention No. 182)

Noting the potential for confusion about what constitutes work likely to harm a child’s health, safety or morals, the ILO passed Recommendation No. 190 to specify such jobs as: (a) work which exposes children to physical, psychological or sexual abuse; (b) work underground, under water, at dangerous heights or in confined spaces; (c) work with dangerous machinery, equipment and tools, or which involves the manual handling or transport of heavy loads; (d) work in an unhealthy environment which may, for example, expose children to hazardous substances, agents or processes or to temperatures, noise levels or vibrations damaging to their health and (e) work under particularly difficult conditions such as work for long hours or during the night or work where the child is unreasonably confined to the premises of the employer. The WFCL Convention itself thus amalgamates elements of what is elsewhere defined in international law as both ‘slavery’ and ‘slavery-like’ conditions. Like the colonial era, this understanding of children’s permissible work spreads from the North to the South but conflicts with contextual conditions and lifestyle of majority of children living there, leading to the creation of hybrid childhoods and distorted perceptions of the children’s reality (Liebel, 2020).

## Child trafficking and its attendant policy measures in Ghana

Ghana’s recent frowning on children’s mobility for work, especially in fishing, follows a ‘new normal’ in international child protection intervention where children’s migration for anything other than schooling is conceptualised as deviant (Boyden & Howard, 2013; Howard, 2017; Okyere, 2022; Timera, 2018). This is in part traceable to Ghana’s ratification of the United Nations Convention on the Rights of Children (UNCRC), and its concomitant legislative and policy framework (which I have discussed in detail in the previous section) on children’s work. Prior to this period, concerns about child labour dominated child protection discourses, amidst a neoliberal Structural Adjustment Programme (SAP) that has been cited as the most critical factor leading to the diminishing of labour standards and the integration of children into the labour markets (Boakye-Boaten, 2010; Lawrance, 2010; Sackey & Overa, 2015). Nonetheless, disagreements between the state, academia and civil society about the permissible degree of work allowed within different socio-cultural contexts means that child labour advocacy failed to achieve much traction. This led to a decline in anti-child labour activism and a consequential rise in anti-trafficking advocacy, following the enactment of the Human Trafficking Act (Act 694) in 2005 (Lawrance, 2010). Legally, proscription of child trafficking appears to have generated fewcontroversies among major actors such as the state, civil society and academia (Dottridge & Feneyrol, 2007; Koomson et al., 2021; Lawrance, 2010). Subsequently, NGOs sought to call out the various dimensions of exploitation and abuse that characterise under-18s’ (especially boys) work in fishing as child trafficking or child slavery (Koomson et al., 2021; Okyere et al., 2021). In framing the practice as child slavery, child rights NGOs are empowered legally to initiate the immediate removal of under-18s from slavery-like conditions. Thus, the use of the term ‘slavery’ is more fashionable and provides a reasonable justification for the conduct of rescue operations by child rights NGOs in Ghana.

Ghana’s anti-trafficking policy is implemented through four main pillars of prevention, prosecution, protection and partnerships (IOM, 2018). Interventions that flow from the policy entails direct assistance to child victims in a two staged approach, namely: (i) transitional assistance (community sensitisation, victim rescue and rehabilitation in a shelter); and (ii) comprehensive medium to long-term assistance which is characterised by the reintegration of child victims and family strengthening through the provision of livelihood support (IOM, 2018). Working closely with state actors such as the Police and Department of Social Welfare (DSW), child rights NGOs remain the key actors in the implementation of child trafficking policy in Ghana. Activities of child rights NGOs who operate within Ghana’s anti-trafficking space are primarily driven by these two approaches. Notable NGOs within this space include Free the Slaves (FTS), International Needs, Challenging Heights and Partners in Community Development Programme (PACODEP). Many of the child rights NGOs secure international funding from the Global North for their field activities by selling the misery and plight of children considered trafficked, through audio-visual campaigns (Okyere et al., 2021) and grant applications. While these NGOs mean well for children considered trafficked, their quest to secure adequate funding means they must adhere to Global North dominated discourses and modalities, consequently undermining contextual factors that inform the problem (Okyere, 2022). In this regard, O’Connell Davidson (2013, 2016) has argued that while charitable acts of child saviourism by NGOs have a part to play in righting child wrongs, it does very little to address the structural determinants of children’s suffering, as children are treated as ‘empty bodies’ without agency and families. In the next section, I discuss how kinship fostering may sometimes be problematised and conflated with child trafficking even though it has been a resilient traditional practice for childcare amidst structural factors that underline children’s economic activities.

## Childhood, kinship fostering and trafficking in the Ghanaian context

Childhood in traditional Ghanaian society is portrayed as a delicate period where children were offered protective covering in the extended family (Boakye-Boaten, 2010). The period is considered as holding an egg in one’s palm, which requires careful attention and protection as holding it tightly may crush it, whilst a careless handling of the egg may also destroy it too (Boakye-Boaten, 2010). Accordingly, children belonged concurrently to a family, kin and community; therefore, child-rearing was a collective responsibility of the family, kin and community (Boakye-Boaten, 2010; Kilbride & Kilbride, 1990). Within this context, children’s work was a considerable part of their socialisation within indigenous economic sectors such fishing and farming. This constitutes the basis of kinship fostering within the Ghanaian socio-cultural context. Kinship fostering provides an alternative care option for children in need of care and protection (Abdullah et al., 2020). It serves as the lifeblood of the traditional Ghanaian family system where extended family members take on parenting responsibilities for relatives who are incapacitated (Manful & Cudjoe, 2018; Nukunya, 2016). In this regard, responsibilities within the families are shared among biological parents and household members when a child is born (Manful & Cudjoe, 2018). As Nukunya (2016, p. 49) puts it, ‘when the mother’s milk is not sufficient, another woman could breastfeed the child. When the mother is not around as the baby cries, another woman cools it down with her breast.’ Consequently, kinship care promotes sustainability and stability in childcare within the family (Manful & Cudjoe, 2018; Nukunya, 2016). Kinship fostering is acknowledged by Ghana’s Child and Family Welfare Policy (2015), even though the Children’s (Amendment) Act, 2016 (Act 937) is silent on the practice. Within the Ghanaian socio-cultural context, kinship fostering entails children’s mobility and placement with an extended family relative in a location that is further apart from their biological parents. Where this is characterised by children’s mobility to fishing communities under the care of a kin, the practice is often flagged as trafficking if evidence exists to suggest that the child in question is involved in work within fishing (Koomson et al., 2021; Okyere, 2022).

However, children’s economic contribution within their household has been a significant part of their socialisation albeit with variations from pre-colonial period through to post-colonial times. For instance, while children worked for family subsistence before colonial rule, the advent of British colonial rule in 1874 introduced several socio-political and economic changes that led to major transformation of the Gold Coast (now Ghana) from a barter system to a monetised economy (Boakye-Boaten, 2010). Consequently, Ghanaian families were compelled to provide cash to pay head tax for members of their households (Sackey & Overa, 2015; Valentine & Revson, 1979). Subsistence practices of agriculture and kinship relations were toppled because of an economy based on cash and on fixed dates of payment. The ensuing social change led to the crumbling of subsistence work for children and the gradual inclusion of children within a capitalist economy promoted by colonial rule. Thus, children were compelled to contribute significantly to the payment of their own head tax through paid work (Sackey & Overa, 2015; Valentine & Revson, 1979). Furthermore, years of political and socio-economic turmoil in the decades following the attainment of independence in 1957 created economic hardships. For instance, the implementation of the Structural Adjustment Program (SAP) from 1983 onwards increased the economic constraints on families in post-colonial times (Bass, 2004), requiring children to contribute economically to their upbringing (Boakye-Boaten, 2010; Sackey & Overa, 2015). The nature and type of work undertaken by children is dependent on the dominant economic activities of their native communities and ranges from work in farming, aquaculture, mining and street hawking. Okyere (2022) observes that children’s work in mining formed part of their attempts to obtain basic life necessities such as obtaining food, accessing healthcare and pursuing development opportunities in school and skills training (Okyere, 2022). Moreover, norms on informal apprenticeship training motivate children’s involvement in Ghana’s cocoa and fishing industry (Sackey & Overa, 2015). While civil society and child protection organisations have labelled children’s work within these sectors as hazardous and exploitative, the academy have differed in perspective.

I argue that the contention between the academy and civil society on children’s work is in part due to the decolonial turn in social and political sciences that challenges the taken-for-granted assumptions that underpin the concept of the universal child. Twum-Danso Imoh (2016) asserts that there has been an overstatement and overemphasis on the marginalised childhood of the South which is mostly characterised by lack, deprivation and constraint. As argued by Boyden, the local realities of children in the South serve to reify its dissonance with the global hegemonic ideal, with its roots in the North. The local is consistently posed in contrast with the ideal. Accordingly, culturally or contextually specific childhoods – such as child-headed households, children’s sexualities, marriage and work expectations – are not regarded as valid or meaningful (Sumberg & Sabates-Wheeler, 2023). Within the discourse of decoloniality, this is referred to as the ‘denial of coevalness’ (Mignolo, 2007); the referencing of global historical transformations to Western experience and the disregard of non-Western childhoods as evolving in a timeframe of its own (Blazek, 2024). However, for child trafficking intervention to achieve its desired purpose, there is the need to pay attention to contextual understanding of childhood, while concurrently retaining a focus on the overarching processes that shape children’s experiences across diverse locations (Blazek, 2024). This is not a far-fetched dream, as child rights NGOs utilise kinship fostering in child reintegration (Hamenoo et al., 2020; Peasah, 2008) even though kinship fostering has been implicated as a tool for the trafficking of children in several studies (Agbenya, 2009; Einarsdottir & Boiro, 2014; Hashim, 2006; UNICEF, 2005).

## Research context and methodology

The ethnographic research that this paper draws upon is situated within the complex context of intersectionality between the Western inspired legal prohibition of children’s mobility for work in fishing, the rescue and reintegration activities of NGOs, and the contradictions that evolve in respect of intent and practical realities. The paper explores the perspectives of young adults (16 years–22 years) and NGO officers on issues of rescue, reintegration, and post-rescue experiences. It examines how the local law on human trafficking (based primarily on the Palermo protocol) leads NGOs to summarily conflate young adults’ economic mobility as trafficking, consequently resulting in their removal from fishing communities along the world’s largest artificial lake, with promises and assurance of a better life within their communities of origin. By employing a critical approach to the analysis of primary (biographical interviews, observations) and secondary data (analysis of legal documents), the appropriation, translation, and remaking of human rights ideas within the study community is fleshed out (Merry, 2006) to indicate how translations of universal human rights language can only be achieved partially, as they continuously engage with locally situated ethics, moral systems, and priorities (Ong, 2009). In this regard Tsing (2004) has proposed the lens of ‘friction’ for an ethnographic study of global interconnections to describe the difficult translations, conjunctures, and contingencies through which universal claims are ‘charged and enacted in the sticky materiality of practical encounters,’ with those encounters recurrently being transformed. While the adoption of this lens is suitable for the study of a social process like Ramachandran’s (2019) study of domestic work and labour trafficking in India, I demonstrate how the promises of antitrafficking rescues and reintegration are not only inflected by socio-cultural and economic factors that exist beyond the influence of NGOs, but are equally affected by specific idiosyncratic beliefs of the subjects of study. Thus, this paper adopts a nuanced stance between the character-driven and process-driven subject categorisations highlighted by Jerolmack and Khan (2017). Moreover, the lens of ‘friction’ provides an appropriate framework for situating the study discussions within the context of decoloniality.

The fieldwork for this article forms part of a much larger project (Modern Marronage Pursuit and Practice of Freedom, MMPPF study 2) which examined the lives of young working adults within fishing and mining communities in Ghana. The field research was conducted between July 2022 and September 2022 within the fishing suburbs of Winneba. Winneba is the district capital of the Effutu Municipality, one of the Central Region’s twenty districts. It remains the only urban settlement in the municipality, with a population of 99,898 people, making up 92.68% of the district’s population (GSS, 2021). The major source of livelihood is craft and related trading activities (31.4% of the working population in this area), while 16.1% are engaged in agriculture, forestry, and the indigenous fishing industry. Productive fishing in Winneba is seasonal as it lasts only for a limited period in the year – from August to September (Sefa-Nyarko, 2016). It is not unusual to see children playing along the beaches and interacting with local fishers who are primarily their own parents, relatives, and acquaintances. Other activities undertaken by children along the beaches include pulling and maintenance of nets, and swimming in the shallow waters for leisure (Koomson & Abdulai, 2021; Sefa-Nyarko, 2016). This builds the competence and skill of children for specific roles within the local fishing industry, making them desirable assistants for fishers on the Volta Lake. The municipality has been cited in several reports as a source community for the trafficking of children to riverine communities in the Volta and Bono East Regions of Ghana respectively (Koomson & Abdulai, 2021; Singleton et al., 2016). As detailed elsewhere by Okyere et al. (2021), the last decade has witnessed rigorous efforts by numerous domestic and foreign anti-trafficking organisations, seeking (through the media) to present and address forms of children’s work in riverine communities as outright forms of child exploitation, child trafficking, and child slavery. The claim has been made through media representations that thousands of children have been bought for as little as USD 20 in Ghanaian coastal areas such as Winneba and trafficked to work for 15 h daily in communities along the Volta Lake with no prospect of escape (Fisher, 2017; Freduah, 2008). These harrowing accounts and portrayals of ‘child slavery’ and ‘trafficking’ on Ghana’s Volta Lake have unavoidably attracted significant international attention and swift response by way of rescues (Okyere et al., 2021). Apart from being the source community, Winneba remains a reintegration destination for young adults who have been rescued by child rights NGOs and their international partners (Koomson & Abdulai, 2021; Sefa-Nyarko, 2016).

Given that the study sought to explore the post-rescue experiences of rescued young adults considered trafficked, a critical ethnographic approach was employed to document their lived experiences five years after reintegration. Thus, a key point of originality and value-addition by this paper to the discourse of rescue and reintegration is to trace the storied narratives of rescued young adults, which is effectively missing in recent post-rescue studies within child trafficking literature. By critical ethnography, I progress from the presentation of young adults’ voices to the questioning of ‘dominant and hegemonic discourses’ (Madison, 2020, p. 30) about rescue and reintegration within anti-trafficking intervention. Little is known about the medium and long-term wellbeing of children who are progressively entering adulthood after reintegration, as extant studies have revolved around issues of reintegration success versus the number of re-trafficking. In so doing, depth is sacrificed for breadth as critical issues of wellbeing such as feeding, shelter, health and parental care are not thoroughly explored. Accordingly, I employed a semi-structured interview guide (flexibly) to explore specific aspects of young adults’ post-rescue lives tracing their daily experiences while in temporal placement within a shelter to the present days of the field activity of this study. In practical terms, I started with a reconnaissance meeting at the office of one of the child rights NGOs, where I had a conversation with a ‘Reintegration Officer’ (NGO Officer-gatekeeper) who provided background knowledge and understanding about his organisation’s rescue and reintegration programme. This preliminary exercise offered the opportunity to obtain specific view of the research participants (young adults) who will be most pertinent to the study. Following this meeting, a probable list of interviewees was shortlisted according to the study’s criteria (experience working with fishers within one of the riverine communities along the Volta Lake; at least five years post-rescue experience living within the study community; and age range between 16 and 22 years). Young adults between the ages of 16 and 22 years were selected because they were well-informed to share their experience of the reintegration process. Moreover, the five-year post-rescue criterion was to ensure that young adults had been completely weaned from NGO intervention and had adequate post-rescue experiences to share. The profile of study participants has been summarised in Table 1 below.

**Table 1.** Profile of participants.

Category	Variable	Frequency	Percentage (100)
Sex of study participants	Male	14	73.7
	Female	5	26.3
Age range of young adults	16–18yrs	5	38.4
	19–20yrs	4	30.8
	20–22yrs	4	30.8
Number of years after reintegration	At most 5yrs	9	69.2
	Above 5yrs	4	30.8
Number of years working for NGO	0–5yrs	2	33.3
	6–10yrs	4	66.7

The NGO officer (my gatekeeper) led me to the study participants residents to explain the purpose of the research, obtain initial consent and assent for the 18-year-olds and under-18s, respectively. Following the signing of the consent forms by the study participants and their parents, specific interview dates, times and venues were scheduled according to the convenience of study participants. While sixteen young adults had expressed interest to participate in the study, I ended up interviewing thirteen of them as they were readily available during the field work. Additionally, notes were taken as observable reflections (Merriam & Tisdell, 2016) while walking within the fishing suburbs of the study community under the guidance of my gatekeeper. Furthermore, six NGO officers (including the gatekeeper) were interviewed. Transcribed interview data was organised into the ‘basic situations of human life’ schematic category suggested by Lofland and Lofland (1995, p. 104), which includes findings about family, education, and health (Merriam & Tisdell, 2016). In so doing, concepts from the interview transcript and those gleaned from the literature on child trafficking intervention (emic perspective) were weaved by the ethnographer (etic perspective) into a critical description of young adults’ post-rescue experience and NGO Officers’ reflections.

## Rescue and reintegration: assessing the concept of freedom

None of the young adults interviewed reported they resisted attempts to be rescued and brought back home to reunite with their parents. Perhaps the eagerness to jump into the boat of the child rights NGO stemmed from the promise of a better life offered by the officers steering the rescue operation, as is mostly the case with rescue campaigns (Okyere et al., 2021). One of the young adults recounted how the mention of ‘freedom from hazardous labour on the lake,’ and a ‘daily life of bliss in the classroom’ enticed him to immediately jump into the boat of individuals he was meeting for the first time in his life. Surprised at his lack of scrutiny, I asked why he did not experience any iota of apprehension when the NGO officers offered to take him home, given he was meeting them for the first time. He indicated that ‘I have heard that they take children back to Winneba for school, so I was always looking out to see that particular boat until they arrived and said my mother has asked them to bring me back.’ The NGO officer who led me to the study participants added that his organisation undertakes targeted rescue operations which entail ‘collaborating with parents or guardians to rescue children who have been trafficked to work in riverine communities, hence rescue teams provide adequate information to targeted victims, including showing them pictures as evidence before they agree to move with them.’ While the officer’s explanation could account for the ease with which the young adult jumped into the rescue boat; the reason for his decision was the promise of a better life very different from his lived experience within the fishing community where he was rescued.

Anti-trafficking campaigners promise a haven for victims of trafficking who are considered entrapped in ‘hell’. The selling point of these campaigns is to portray specific social contexts as havens, using disturbing images about other contexts which are considered hazardous for the development of children (Okyere et al., 2021). In placing this rationale under the critical lens of the lived experiences of rescued young adults, I explored the storied account of their rescue journey and discovered that their post-rescue experience is not necessarily romantic as they were made to believe, as it was still characterised by exploitative work, residential insecurities, parental separation, and everyday violence. Kojo’s story was of particular interest to me and central to this paper because of his ‘darling’ status. Kojo’s ‘darling’ status within the NGO’s rescue programme stemmed from the fact that he had been a victim of re-trafficking according to the NGO officers I interviewed. When I asked for the best participants to recruit for this research, my field guide (NGO officer) happily led me to Kojo.

Kojo recounted how he had been initially taken to Yeji (the fishing community along the Volta Lake) by his mother to live and work with his mother’s sister and her husband (fisher) as his mother could not provide for him and his five siblings following a separation between his biological parents. After living and working for almost five years, he was rescued by a child rights NGO and brought back home to live with his mother after he had spent three months in a shelter in the Central region of Ghana for rehabilitation. However, Kojo indicated that

my mother could not adequately take care of me and my siblings when support from the NGO ceased after two years. Consequently, my mother advised me to go and live with her friend in Accra, who had promised to train me in school.

When Kojo got to Accra with his mother, he realised his mother’s friend lived in Yeji and was taking him to Yeji to live with him. Given Kojo’s previous experience in Yeji and numerous lessons received from NGO officials on the tactics of traffickers, he indicated that ‘I raised an alarm by shouting that I have been sold to my mother’s friend.’ Asked why he did that, Kojo responded that ‘during my first post-rescue experience at the NGO’s shelter, we were told to do so anytime we felt we were being transported to a place we had less information about.’ Following the arrest of Kojo’s mother and her friend, Kojo was placed in a shelter in Accra and used as a prosecuting witness in a case that lasted for a year until his mother and her friend were jailed for five years respectively. Asked about his living experience in the shelter, Kojo raved about the living conditions at both shelters as follows:

during my first shelter experience, I enjoyed my stay there because we do not work like I used to do when I was living with my mother’s sister at Yeji. I only wake at 5:00am, sweep my allotted portion and prepare for my breakfast and class. I was eating three times daily with snacks, and I had the opportunity to play, watch movies and receive gifts.

Asked about the second shelter experience in Accra, Kojo indicated that: ‘I liked the place in Accra as well, I was going to school, and I had all my basic needs. I was even made a chaplain in the two years that I lived there.’ The romanticised conditions of both Shelters were corroborated by all the young adults interviewed. For instance, Kweku, a reintegrated young adult, rhapsodised about how he had tried ‘unsuccessfully to convince the NGO officer to take him back to the shelter since he missed the place.’ It appears that the promise of a blissful life after rescue was fulfilled to young adults, albeit for a brief period.

While the lived experiences of Kojo and other young adults were far from rosy while they lived and worked within some of Ghana’s riverine communities; are they necessarily free from exploitation, harm, and abuse after their reintegration? Answers to these questions were complex, stemming from dissimilar, often conflicting perspectives on socioeconomic realities, proper family life, and legal opinions. Nonetheless, I show how Kojo and other study participants’ life of bliss in the shelter vaporised into thin air after they had been reintegrated.

In reference to Kojo’s second reintegration, he consistently battled with residential instability. Following the incarceration of his mother and her accomplices in accordance with Ghana’s Human Trafficking Act, 2005 (Act 694), Kojo’s options of a place called ‘home’ were limited. Within four years he had lived with four different guardians. Kojo gave the account of his post-rescued experience as follows:

When I returned to Winneba after the court case involving my mother, I had to stay with an elderly family friend whom I considered as my foster grandfather. While living with him, life became difficult after we tried unsuccessfully to do other businesses. I had to move and live with a man who sold tap water. After living with him for three years I was kicked out for insisting that I would want to go back to school. So, I moved to stay with one of our church members and it was there that I realised that I had contracted tuberculosis, so he could not live with me again. I had no other option than to move out and stay with my pastor who took care of me when I contracted tuberculosis. I returned to my foster grandfather after I had recovered from the tuberculosis infection.

Asked why he was not placed with his biological father or mother’s family relatives, Kojo indicated that ‘my role as a prosecuting witness in my mother’s incarceration had given me a “bad boy” tag within my extended family where I could have easily returned to’. Accordingly, Kojo can be said to have been stigmatised and ostracised by his kin.

Aspects of Ama’s post-rescue experience mimic that of Kojo’s experience. She recounted how her biological mother was denied an opportunity to take care of her under the pretext of her mother’s irresponsibility, indicating that: ‘I was first placed with my mother’s sister. However, while living with her the support from the NGO expired and things were hard, so I struggled. The NGO spoke to my father, and I was moved to live with my father’s sister since my father has travelled.’ Ama’s struggles to find a suitable place to live are all the more puzzling if one considers the fact that her biological mother is living very close by. She expressed frustrations with her biological mother’s reluctance to accommodate her because her biological mother claimed that ‘the NGO has total control over your life since the NGO brought you back from Yeji at the request of your father.’ As I was curious to understand why Ama’s biological mother had been given limited access to her daughter, the NGO officer who had led me to the study participants explained that her mother’s ‘socio-economic circumstances had rendered her incapable of providing proper care for her daughter.’

Another crucial issue that characterises young adult’s lives is their post-rescue economic activities. Given that abolitionist rhetoric hinges on the vulnerability of children and the peril of further exacerbating this vulnerability through their incorporation into specific economic activities considered hazardous and exploitative, schooling is considered the most appropriate alternative as it offers children the opportunity to learn until they turn 18 (Boyden & Howard, 2013; Howard, 2017; Timera, 2018). Consequently, one would expect that children rescued from hazardous and exploitative work would as well be protected from potentially engaging those precarious economic arenas, and instead remain in school. However, experiences of some of the young adult participants in this study indicated otherwise. Young adults like Kojo had to work to survive, which included staying out of school and working for long hours. Kojo narrated his experience as follows:

When I lived with my foster grandfather, he bought bags of sachet water and stocked them in the fridge so I could sell them after school every day to support myself. Nonetheless, the refrigerator broke down, so things became difficult for me.

Following the collapse of the water business, Kojo turned to other menial jobs until he received a proposal from a man who owned and operated a commercial tap for selling water. After Kojo’s foster grandfather had successfully negotiated with the owner of the tap water, Kojo started working by selling tap water within his community*.* Nevertheless, Kojo indicated that he was not ‘going to school regularly since he started selling the water at 4:30am and closed at 8pm at the earliest’. The strenuous nature of Kojo’s work schedule was also exacerbated by the fact that he had to support his employer’s wife in her house chores. Another young adult, Adwoa, recounted how she would have to forego specific schooling days in the week to assist her father’s sister in selling. This, according to her, ‘affected my academic performance and I was sometimes whipped in school for my truancy.’ When asked if her father’s sister was aware of the effect of her truancy, Adwoa indicated that she had complained several times but her father’s sister abused her verbally by referencing the ‘irresponsibility of her biological mother and father.’ Moreover, Kwame, another young adult participant, noted how he had to ‘sell yoghurt after school and sometimes during school hours’ to provide for himself since his guardian could not adequately do that. While one of the young adults, Kofi, indicated that his apprenticeship training provided avenues for him to ‘cater for himself through the tips offered by his master,’ he, like other young adults in apprenticeship, had to occasionally work outside of his trade domain as well. Accordingly, the post-rescue experiences of the young adults in this study required them to engage in some economic activities in order to survive.

## Rescues as solutions to child trafficking/slavery: *towards a critical discussion*

The promise of a post-rescue bliss for young adults by NGOs, and the paradoxical realities of young adults, suggest the need to understand rescue and reintegration in terms of the socioeconomic conditions and cultural practices that frame children’s work in fishing. The paper’s intention is not to condemn the removal of minors from economic activities that pose as risk to their health and development, but rather, to underscore its limitations as a solution, while suggesting other viable alternatives.

In the shelter, young adults recounted how the NGO’s promise of a blissful experience was seen in full force. The romantic childhood experience that NGOs promote however seems to be realistic only within a confined shelter environment where all exogenous factors can be controlled. Frimpong-Manso and Bugyei (2019) have observed that financial constraints, inadequate preparations, and the lack of consultation with children underline the stated difficulties in replicating the shelter environment at home for reintegrated young adults. Under such circumstances, minimal support from NGOs or relevant social services may worsen the plight of reintegrated individuals. Given that NGO resources are limited in supply and tied to specific timelines (Wedge et al., 2013), it is erroneous to assume that the mere reunification of victims with their families may be the best option for them. While young adults’ economic migration is mostly plagued with several risks, the practice of removing and reunifying them with their families is more complicated in its socio-cultural, economic and legal specificities.

At the time of this ethnographic research, the urgency of local NGOs and their donor partners to rescue children considered as trafficked was still fashionable, with some abolitionists citing poor living conditions when I had an opportunity to attend one of their training workshops. The issue of victims’ sleeping duration and location (sometimes in canoes at night on the lake) while reported extensively by studies (ILO/IPEC, 2013; Singleton et al., 2016), was prominent among the factors mentioned by the workshop participants for the necessity of rescues. Nonetheless, some of the young adults who had been reintegrated by the NGO experienced difficulties securing a place to sleep, as evident in Kojo’s ordeal. While a Romanian study found that reunified children were not able to cope with the poor living conditions in their family homes leading to their re-institutionalisation (Bejenaru & Tucker, 2017), results from this ethnographic study have shown how young adults’ lives have been characterised by residential instability because they could not return to the shelter. Moreover, Frimpong-Manso and Bugyei’s (2019) study of reintegratedorphans in Ghana revealed poor living conditions at home and lack of basic amenities such as electricity and pipe-borne water. Basically, reintegrated orphans had difficulty adapting to these conditions as they were used to having them readily available at the orphanage. Reintegrated orphans compared their living conditions with their privileged life at the orphanage, and wished they were still at the orphanage. By implication, the realities of comparing their reintegrated experience with their residential home experience meant that the young adults who were struggling with residential stability were consistently looking out to find a home which would bear some similarities to the shelter. This can be attributed to the weakness in the western-centric romantic logic that underlies childhood, which seeks to impute neoliberal child-rearing ideals on African childhood. On the one hand, child rights NGOs often construct child placement with extended family relatives as undeniable evidence of child trafficking; especially where this involves work (Berlan, 2009; Koomson et al., 2021)^2017^ since neoliberal ideals promote two-parent-led nuclear families as the optimal or legitimate family model for children’s upbringing (Cooper, ). On the other hand, children are placed with extended family relatives in post-recue programmes by child rights NGOs on the basis that they will be well catered for. This reflects the post-colonial connections and contradictions between the global and local dimensions of childhood and the lifestyles of children on the objective and subjective level (Liebel, 2020).

On a related note, rescues foregrounded as a global antitrafficking response assume that the best way to sustain the freedom of rescued victims lies in the swift arrest and incarceration of those considered as traffickers. Kojo’s peculiar role as a prosecuting witness presents a conflicting perspective of how a well-intended act of deterrence and protection may further exacerbate the vulnerability of those they are intending to protect. While the number of prosecutions has not commensurately reflected the number of rescues (Okyere et al., 2021; Sertich & Heermskerk, 2011), the central case presented in this ethnographic research is indicative of how autochthonous social-cultural beliefs (Lawrance, 2010) may hinder successful application of Ghana’s Human Trafficking Act, 2005 (Act 694) which remains a carbon copy of the Palermo Protocol (Sertich & Heermskerk, 2011). For example, Kojo’s exercise of his right under the Human Trafficking Act, 2005 (Act 694) was interpretated as deviant by members of his extended family mirrors the definitional and contextual problems that characterise the Act. Studies (Andrew & Lawrance, 2012; O’Connell Davidson, 2013; Sertich & Heermskerk, 2011) have drawn attention to the problems of definitional and contextual clarity in respect of anti-trafficking legislation. Specifically, Andrew and Lawrance (2012) have bemoaned the acontextual feature of the Human Trafficking Act, 2005 (Act 694), and its consequential ambiguity. Evidently, provisions of the Act are at variance with the beliefs of Kojo’s extended family relatives who expected him to be a ‘good child’ (Kirby, 2020) by declining to testify against his mother in court. Thus, contradictions and tension between antitrafficking laws and their contextual application (Ramachandran, 2019) are evident in this context. For Kojo, strategies arising out of the Act’s application had led to a separation between him and his family, making reintegration into his natal community a Herculean task. In such circumstances, the vulnerabilities of rescued children are rather exacerbated and complexified, leaving them susceptible to re-trafficking as observed by Golo and Eshun (2019) in their study on re-trafficking within fishing communities.

The fact that most young adults had to work to survive further reinforced the contradictions that characterise anti-trafficking policy, and in this regard rescues and post-rescue interventions. This finding is even more informative if one considers the fact that young adults’ economic activities had to compete with their schooling days. Studies have shown that reintegrated children experience challenges with their education, while some even drop out of school, which raises the risk of child labour and marriage (Save the Children, 2004). Moreover, due to their caregivers’ limited resources, most reintegrated children were working to obtain the resources needed for school according to Frimpong-Manso and Bugyei’s (2019) study of reintegrated orphans in Ghana. Others had even stopped schooling and were engaged in factory employment. The trade-off between work and schooling for reintegrated children was affirmed by a study of reintegrated victims of child trafficking in Ghana. In particular, the study (Hamenoo et al., 2020) found that reintegrated children were engaged in work (fishing and farming) that can be classified as hazardous by Ghana’s Children’s Act, 1998 (Act 560). This finding erodes the intended purpose of rescues, rehabilitation, and reintegration, while affirming the sheer exclusivity of the socio-economic factors that underline under-18s’ mobility for work. It further questions the logic of rescues being an end in themselves, since young adults in this study returned to the precarious conditions they were supposedly rescued from. Analysed more broadly in terms of a post-colonial literature on childhood, I argue that the assumptions of rescue are driven from and dominated by Western interests and ideals (Abebe, 2019; Koomson & Abdulai, 2021; Twum-Danso, 2016). Thus, Western donors’ pressure on Ghana to adopt and assimilate its child rights governance and practice regime consequently empowers aligning NGOs to undertake rescues and reintegration interventions that fail to consider the sociocultural and economic context within which they operate. In the words of Mutua (2016), this flows from an international human rights order that sees deficits in Global South practices.

## Conclusion

This ethnographic research has contextualised the legal and practical realities that characterise post-rescue interventions. For NGOs pursuing an antitrafficking agenda through rescue and reintegration of children considered trafficked, laws represent important instruments that legitimise their intervention. The application of these laws, however, is shaped by inconsistencies and ambiguities inherent within legal definitions and provisions, as well as the prevailing socioeconomic and cultural context within which the laws are implemented. This research has outlined the socio-legal context within which a long-standing culture of children’s and young adults’ work in fishing coexists uneasily with donor-driven NGO rescues and reintegration. This complex terrain of frictional realities is not necessarily a product of contradiction between the Ghanaian legal provision and its derivative international conventions, but rather, an alienation of the Ghanaian law from its contextual realities. Consequently, the intended purpose of rescues and post-rescue interventions, I argue, seems to be a far-off dream or at least an impractical reality since beneficiaries are not necessarily freed from the difficulties and challenges that characterised their pre-rescued lives.

This research, however, presents useful insights that can serve as starting points for child rights and antitrafficking intervention. In terms of the legal provisions that relate to the conceptualisation of childhood, it is imperative to de-colonise those provisions by reviewing and amending specific aspects of the Children’s Act, 1998 (Act 560) and Human Trafficking Act, 2005 (Act 694) that veil children’s agency and criminalise their involvement in specific economic activities such as freshwater fishing. Given the fact that the stated reasons for their removal from these activities continue to persist for a considerable number of them after reintegration (Mbamba et al., 2023), it is only logical to switch the focus of anti-trafficking legislation from a quick-fix protective purpose to a medium- to long-term protective solution in practice. This calls for legislative provisions that will precipitate better policy initiatives such as providing teenagers who desire to go into those occupations with protective mechanisms. This will serve them better than eradicating the possibility of their employment altogether. Additionally, clearly defined rules of duration of work for under-18s and rewards can be introduced to avert exploitation and abuse. This is workable because studies (Koomson et al., 2021; Nieuwenhuys, 1994) have shown that most experienced fishers enter the trade during the start of their teen years.

Finally, in the cases I have observed in this ethnographic research, rescues temporarily addressed one aspect of the larger sociocultural underpinning childcare in Ghana. The placement of young adults with foster families and other relatives did not necessarily erase their vulnerabilities, but rather diversified the vulnerabilities of some of them. How, if at all, can intervention by NGOs address the vulnerabilities of rescued young adults? Removal of young adults from vulnerable environments, while well-intentioned (Mbamba et al., 2022), does not necessarily constitute a magic bullet; it is imperative to focus on strengthening the traditional institution of kinship care/fostering through the provision of professional social service such as monitoring. Fostering remains a viable alternative for parents who cannot adequately cater for their children in Ghana; consequently, the conflation of fostering with trafficking has only succeeded in demonising a cultural practice that has persisted for ages in the absence of an efficient formal social care (Koomson et al., 2021). Additionally, the fact that most of the young adults in this study were placed with extended family relatives emphasizes the need to address the contemporary weakness of kinship fostering. For instance, families who provide foster care may be given priority in the Livelihood Empowerment Program Against Poverty (LEAP). This will reduce the vulnerability of persons in foster care as they would not compete with other children for the limited family resources, as studies (Fernandez et al., 2017; Frimpong-Manso & Bugyei, 2019) have shown.
